# Real-world results of autologous stem cell transplantation in newly diagnosed multiple myeloma: a report from the Canadian Myeloma Research Group database

**DOI:** 10.1038/s41408-023-00905-8

**Published:** 2023-09-05

**Authors:** Julie Côté, Richard LeBlanc, Hira Mian, Michael P. Chu, Arleigh McCurdy, Esther Masih-Khan, Jiandong Su, Victor H. Jimenez-Zepeda, Kevin Song, Martha Louzada, Darrell White, Michael Sebag, Anthony Reiman, Julie Stakiw, Rami Kotb, Debra Bergstrom, Muhammad Aslam, Rayan Kaedbey, Christopher P. Venner, Engin Gul, Donna Reece

**Affiliations:** 1 CHU de Québec—Université Laval, Quebec, QC Canada; 2 Hôpital Maisonneuve-Rosemont, Montreal, QC Canada; 3 Department of Oncology, McMaster University, Hamilton, ON Canada; 4 Department of Oncology, Cross Cancer Institute, Edmonton, AB Canada; 5 Department of Medicine, Division of Hematology, The Ottawa Hospital, Ottawa, ON Canada; 6 Department of Medical Oncology and Hematology, Princess Margaret Cancer Centre, Toronto, ON Canada; 7 Canadian Myeloma Research Group, Toronto, Ontario Canada; 8 Arnie Charbonneau Cancer Institute, University of Calgary, Calgary, AB Canada; 9 Division of Hematology, University of British Columbia and Leukemia/BMT Program of BC, Vancouver General Hospital, Vancouver, BC Canada; 10 University of Western Ontario, London Health Sciences Centre, London, ON Canada; 11 Division of Hematology, Dalhousie University and Queen Elizabeth II Health Sciences Centre, Halifax, NS Canada; 12 Division of Hematology, McGill University Health Centre, Montreal, QC Canada; 13 Saint John Regional Hospital, Saint John, NB Canada; 14 Saskatoon Cancer Centre, Saskatoon, SK Canada; 15 Department of Medical Oncology and Hematology, CancerCare Manitoba, Winnipeg, MB Canada; 16 Division of Hematology, Memorial University of Newfoundland, Newfoundland and Labrador, St John’s, NL Canada; 17 Allan Blair Cancer Center, Regina, SK Canada; 18 Jewish General Hospital, McGill University, Montreal, QC Canada; 19 Lymphoma and Myeloma Program, BC Cancer, Vancouver Centre, Vancouver, BC Canada; 20 Canadian Myeloma Research Group, Toronto, ON Canada; 21 Princess Margaret Cancer Centre, Toronto, ON Canada

**Keywords:** Myeloma, Myeloma

## Abstract

Autologous stem cell transplant (ASCT) remains an important option for eligible multiple myeloma (MM) patients as part of initial therapy. Using the Canadian Myeloma Research Group (CMRG) national database, we examined the details and outcomes of ASCT performed as first-line therapy in eligible Canadian MM patients between 2007 to 2021. We included 3821 patients with 72% receiving CyBorD induction and 2061 patients receiving maintenance, consisting of lenalidomide +/- steroids in 78.3%. The median PFS and OS for patients given a single ASCT were 35.4 and 126 months. Those receiving a second induction regimen had significantly inferior outcomes, although when maintenance was used, results were comparable regardless of the number of induction regimens administered (median PFS 55.3 vs 51.1 months [*p* = 0.11]; median OS 158.6 vs not yet reached [*p* = 0.13]). Consolidation patients had a longer median PFS (55.3 vs 34.4 months [*p* = 0.001]), but no significant gain in median OS (*p* = 0.065). Patients who received lenalidomide-based maintenance experienced a median PFS of 53.7 months and OS of 159 months. In the multivariable analysis, use of any type of maintenance therapy vs no maintenance was associated with a lower risk of progression (HR 0.52 (95% CI 0.47-0.57)) and death (HR 0.58 (95% CI 0.51-0.67)). This real-world study demonstrates that, overall, first-line treatment sequence in transplant-eligible patients produces a median OS of ≥10 years. It also highlights the contribution of post-ASCT maintenance, particularly lenalidomide given until progression.

## Introduction

Multiple myeloma (MM) is an incurable plasma cell neoplasm. Success in treatment of MM focuses on obtaining deep and durable responses which show a positive impact both on progression-free (PFS) and overall (OS) survival. In first-line therapy, autologous stem cell transplant (ASCT) remains the best option for transplant-eligible MM patients [1]. In recent years, ASCT therapy has evolved into a carefully crafted combination of pre- and post-treatments, namely induction, single or tandem ASCT, consolidation and maintenance. The introduction of novel classes of drugs, such as proteosome inhibitors (PIs) and immunomodulators (IMiDs) used in induction, consolidation or maintenance therapy has led to deeper responses [2].

In Canada, the national standard involves a course of bortezomib-based induction. Tandem ASCT is considered for patients with high-risk MM as standard-risk MM patients routinely receive a single ASCT. To further deepen responses after frontline ASCTs, limited durations of consolidation treatments post-ASCT prior initiation of maintenance have also been explored but are perhaps more controversial in their outcomes [3]. Public funding for consolidation has been limited in Canada as well.

Current standard practice for treating newly diagnosed patients after ASCT is the use of continuous maintenance therapy. Trials evaluating lenalidomide maintenance therapy in this context have shown a clear PFS benefit [4–6]. One of those trials also showed an OS benefit [4]. A meta-analysis confirmed an OS gain with lenalidomide maintenance [7]. A previous retrospective analysis from the CMRG also shown, in the real-world setting, a positive impact of lenalidomide maintenance on PFS and OS [8]. Maintenance therapy after ASCT in first-line treatment has been recommended in Canada, and since 2013, lenalidomide maintenance until disease progression has been supported by public funding, although some earlier patients received lenalidomide or other agents via alternative funding mechanisms. However, many real-world patients do not meet the stringent criteria of clinical trials such as those described above, and long-term Canadian data demonstrating the utility of this costly combination therapy approach has been lacking. As such, an analysis on the survival impact of different transplant approaches, including maintenance, used in the upfront setting in the Canadian landscape is of critical importance.

Using a large national retrospective database, our study aimed to assess the outcomes of first-line therapy among newly diagnosed MM patients receiving ASCT. The details of the regimens used for the different components of the transplant sequence (induction, consolidation, and maintenance) as well as their contribution to clinical outcomes were defined. In addition, the results of tandem ASCT, particularly in high-risk MM patients, was evaluated. Knowledge of this information will allow physicians to better understand the different components of the transplant process when performed as part of initial therapy in eligible patients. In addition, given the rapid identification of new drugs and combinations, our results provide benchmarks for comparison when these therapies are introduced.

## Methods

The Canadian Myeloma Research Group (CMRG) is a Canada-wide network of researchers, from 16 major Canadian institutions. The national CMRG database, now consisting of over 9000 patients, is a web-based centralized platform which contains both legacy and ongoing prospective data and can track and characterize real-world outcomes of MM patients treated at the different active CMRG database sites across Canada. All participants have provided informed consent to collect their data as per REB requirements.

This is a retrospective cohort study utilizing the national CMRG database. All patients who received an ASCT as frontline therapy for MM from 1-Jan-2007 to 1-Dec-2021 were included. Patients <18 years old at time of diagnosis were excluded as well as those who received an induction regimen that was not followed by a ASCT for any reason.

Data collection and analysis ended at our pre-specified study completion date, date of last follow-up, discontinuation of therapy for any reason, or death; whichever comes first.

### Objectives and definitions of study endpoints

The primary objectives aimed to better define the details and outcome of ASCT as first-line treatment in MM patients. Demographic and clinical characteristics of all patients that received frontline ASCT were delineated. For the different components of the transplant treatment sequence, the following features were assessed: (1) types, frequencies, and response rates of induction regimens and, if given, the second-line induction regimens; (2) types and frequencies of consolidation post-ASCT, if utilized (consolidation was defined as 2 to 4 cycles of full dose chemotherapy given post-ASCT prior to a lower dose maintenance regimen); (3) types and frequencies of maintenance, particularly lenalidomide, including the duration and the reason for discontinuation; (4) number of tandem ASCTs. PFS and OS were assessed for the different subgroups. Secondary objectives were to analyze factors influencing long-term survival or risk factors associated with shorter survivals in patients who received frontline ASCT therapy. This study did not report on any individual adverse events due to limitations of the data and its retrospective nature.

PFS was calculated from the time of first ASCT until disease progression, death due to any cause or last follow-up, whichever comes first, while OS was defined as the time from ASCT to the date of death or last follow-up. Overall response rate (ORR) was defined as the percentage of patients with a confirmed CR, VGPR or PR as per [modified] International Myeloma Working Group (IMWG) Uniform Response Criteria for Multiple Myeloma [9]. Patients with unknown or missing response were not included in the analysis or included as a separate category. Duration of treatment (DOT) was defined as the length of time between the date of starting a regimen until the date of last dose of the same regimen. Risk characterization was based on fluorescent in situ hybridization (FISH) cytogenetic studies. High-risk was defined by the presence of any one or combination of del17p, t(4:14), t(14:16). Standard-risk was defined by the absence of del17p, t(4:14) or t(14:16).

### Statistical analysis

Statistical analyses were performed using R core team 2020 (R-4.1.1). All *P*-values were 2-sided and for the statistical analyses, and *p* < 0.05 was considered to indicate a statistically significant result.

Descriptive statistic was used to report baseline characteristics of all transplant-eligible MM patients in this study. Confidence intervals were estimated at (95%). Categorical variables were summarized with counts and percentages. Continuous variables were summarized with means, standard deviation and / or medians, ranges (as appropriate). Chi-squared tests was used to determine differences in baseline and outcome variables among categorical variables.

Time-to-event analyses was used to assess the time from induction to ASCT, PFS and OS. Survival curves were constructed according to the Kaplan-Meier method and impact of covariates of interest were assessed using the log rank test.

Univariate and multivariable Cox regressions were performed to explore the impact of maintenance therapy on the disease progression and overall death, controlling for patient characteristics, disease cytogenetic types, stages, as well as laboratory tests. Step down variable selection method was used, Akaike Information Criterion (AIC) was treated as the reference of model fitting statistics.

The following variables were included in the analyses: sex, age, monoclonal protein isotype, ISS stage, laboratory parameters (hemoglobin, platelet count, beta 2-microglobulin, serum albumin, calcium, LDH, FISH cytogenetic subgroup, 1 vs ≥2 induction regimens, single vs tandem ASCT, consolidation therapy (yes vs no) and maintenance therapy (yes vs no)). The R-ISS staging was not entered into regression since it has high collinearity with ISS staging, and it contained more missing values. In the multivariable analysis, some variables were selected out by step-down variable selection method or clinical plausibility.

This study was approved by the Research Ethic Board of Princess Margaret Cancer Centre, Toronto, Canada. Only aggregate patient data is shared outside the database.

## Results

Overall, at the time of data extraction, there were 8600 patients in the CMRG database. 4713 patients started first-line treatment as induction with intent for ASCT. Patients who did not eventually receive ASCT were removed (442 patients). Only patients who commenced first-line induction therapy between January 2007 and December 2021 were included, leaving 3821 patients for the analysis.

### Population characteristics

Baseline characteristics of the 3821 patients were stratified by single or tandem ASCT and are shown in Table 1. Overall, the median age was 61 years, and the majority were male. Race or ethnicity was not available. Sixteen percent of the patients presented with significant renal insufficiency (serum creatinine >177 umol/L). The median time from start of the induction therapy to ASCT was 5.6 months (25–75 percentile: 4.8 to 6.8 months). Most patients received a single ASCT (92%), while 8% received a tandem ASCT. The single and tandem ASCT groups were well balanced except for elevated LDH, higher median beta 2-microglobulin value, ISS/R-ISS stage III and high-risk cytogenetics which were, respectively, more frequent in the tandem ASCT group. Among the entire population, cytogenetic risk data was unknown or missing for 49.8% of the patients. For those of whom cytogenetic risk data was available, 36.7% were high-risk FISH cytogenetics and 63.3% were standard-risk. The majority of patients who underwent tandem ASCT (84%) had documented high-risk cytogenetics.

**Table 1:** Baseline characteristics of the cohort.

Characteristic	Single ASCT (*n* = 3507)	Tandem ASCT (*n* = 314)	Total (*n* = 3821)
**Male sex,** ***n*** **(%)**	2054 (58.6%)	192 (61.1%)	2246 (58.8%)
**Age at treatment initiation, median (range)**	61 (26-77)	60 (26-72)	61 (26-77)
**Myeloma isotype,** ***n*** **(%)**			
IgG	2067 (63.2%)	188 (61.6%)	2255 (63.1%)
IgA	677 (20.7%)	80 (26.2%)	757 (21.2%)
Light chain only	474 (14.5%)	29 (9.5%)	503 (14.1%)
Others	53 (1.6%)	8 (2.7%)	61 (1.6%)
Unknown or missing, *n*	236	9	245
**ISS Staging**			
Stage I	1105 (36.4%)	79 (29.9%)	1184 (35.9%)
Stage II	1074 (35.4%)	83 (31.4%)	1157 (35.0%)
Stage III	859 (28.2%)	102 (38.7%)	961 (29.1%)
Unknown or missing, *n*	469	50	519
**R-ISS Staging**			
Stage I	630 (29.6%)	44 (20.2%)	674 (28.7%)
Stage II	1290 (60.6%)	132 (60.6%)	1422 (60.6%)
Stage III	208 (9.8%)	42 (19.4%)	250 (10.7%)
Unknown or missing, *n*	1379	96	1475
**Hemoglobin, g/L, median (range)**	107.0 (27.0-173.0)	100.5 (39.0-164.0)	106.0 (27.0-173.0)
**Platelet count, 10**^**9**^**/L, median (range)**	223.0 (2.8-977.0)	199.0 (5.0-6.7.0)	220 (2.8-977.0)
**Beta 2-microglobulin, nmol/L, median (range)**	288.0 (20.9-9164.0)	352.4 (85.6-3698.0)	291.7 (20.9-9164.0)
**Albumin, g/L, median (range)**	37.0 (8.0-54.0)	37.0 (14.0-53.0)	37.0 (8.0-54.0)
**Calcium, mmol/L, median (range)**	2.39 (1.23-6.37)	2.4 (1.22-4.6)	2.38 (1.23-6.37)
**Creatinine** > **177 umol/L,** ***n*** **(%)**	522 (16.2%)	60 (20.7%)	582 (16.6%)
Unknown or missing, *n*	294	24	318
**Elevated LDH**, > 250 **U/L,** ***n*** **(%)**	587 (25.2%)	92 (36.7%)	679 (26.3%)
Unknown or missing, *n*	1175	63	1238
**FISH cytogenetics,** ***n*** **(%)**			
**t(4;14)**	240 (11.0%)	87 (39.4%)	327 (13.6%)
Unknown or missing, *n*	1331	93	1424
**17p**	238 (10.6%)	91 (40.8%)	329 (13.3%)
Unknown or missing, *n*	1261	91	1352
t**(14;16)**	63 (4.1%)	29 (21.8%)	92 (5.5%)
Unknown or missing, *n*	1971	181	2152
**Cytogenetic risk classification,** ***n*** **(%)**			
High-risk*	503 (29.9%)	201 (84.5%)	704 (36.7%)
Standard-risk	1178 (70.1%)	37 (15.5%)	1215 (63.3%)
Unknown or missing, *n*	1826	76	1902

*High-risk: presence by FISH of any one or combination of del17p, t(4;14) and t(14;16).

### Induction therapy and ASCT

The majority of the 3821 patients (82%) received a bortezomib-based induction regimen, primarily CyBorD (cyclophosphamide, bortezomib and dexamethasone), in 72.1% (Table 2). Only 1.6% of the patients received an induction therapy with combination of a proteasome inhibitor (PI) and an immunomodulatory derivative (IMiD). For the most frequently used induction regimen, namely CyBorD, the ORR was 92% with 60.4% achieving ≥VGPR. Overall, for the entire cohort, the ORR was 90.7%, with a ≥VGPR rate of 55.5%.

**Table 2:** First induction regimens frequency and response rates.

First induction regimen	Frequency of utilization, *n* (%)	Response rates		
	(*n* = 3821)	Evaluable patients, *n* (%) (*n* = 3444)	ORR, *n* (%)	≥VGPR, *n* (%)
CyBor-D/P	2755 (72.1)	2567 (74.5)	2361 (92)	1550 (60.4)
V/VD	375 (9.8)	321 (9.3)	294 (91.6)	154 (48)
Dexamethasone	339 (8.9)	257 (7.5)	206 (80.2)	74 (28.8)
VAD	99 (2.6)	72 (2.1)	58 (80.6)	14 (19.4)
Other	80 (2.1)	70 (2)	60 (85.7)	37 (52.9)
TD/P	71 (1.9)	62 (1.8)	54 (87.1)	29 (46.8)
PI+IMiD (IxaRD, KRD, RVD, VTD)	62 (1.6)	57 (1.7)	55 (96.5)	36 (63.2)
R/RD	40 (1)	38 (1.1)	35 (92.1)	16 (42.1)

*CyBor-D/P* cyclophosphamide + bortezomib + dexamethasone or prednisone, *V* bortezomib, *VAD* vincristine + doxorubicin + dexamethasone, *T* thalidomide, *Ixa* ixazomib, *K* carfilzomib, *R* lenalidomide, *PI* proteasome inhibitor, *IMiD* immunomodulator.

Because of suboptimal response or progression on first induction, and based on physician decision, 10% of the patients received a second induction regimen before proceeding to ASCT (Table 3). Only four of them needed a third induction (4/376; 1%). The median time from the start of the second induction regimen to ASCT was 4.2 months (25–75 percentile: 3.1–5.7 months). Lenalidomide-based regimens were administered to 73.4% of the patients in this subgroup and resulted, as a second-line induction regimen, in an ORR of 78.6% and ≥VGPR rate of 38.4%. Regardless of the regimen used, patients treated with a second induction regimen had an ORR of 72.9% and a ≥VGPR rate of 35.4%.

**Table 3:** Second induction regimens frequency and response rates.

Second induction regimen	Frequency of utilization, *n* (%) (*n* = 376)	Response rates	
		ORR, *n* (%)	≥VGPR, *n* (%)
CyBor-D/P	33 (8.8)	19 (57.6)	14 (42.4)
R/RD/RP	116 (30.9)	84 (72.4)	45 (38.8)
RVD/RVP	114 (30.3)	93 (81.6)	31 (27.2)
Other	38 (10.1)	20 (52.6)	7 (18.4)
V/VD	21 (5.6)	14 (66.7)	6 (28.6)
KRD	20 (5.3)	20 (100)	15 (75)
DaraRD	16 (4.3)	15 (93.8)	12 (75)
IxaRD	10 (2.7)	5 (50)	3 (30)
TC/TCD/TCP	8 (2.1)	4 (50)	0 (0)

*CyBor-D/P* cyclophosphamide + bortezomib + dexamethasone or prednisone, *R* lenalidomide, *V* bortezomib, *K* carfilzomib, *Dara* daratumumab, *Ixa* ixazomib, *T* thalidomide, *C* cyclophosphamide.

For evaluable patients, the ORR and ≥VGPR rate were 96.4% and 81.1%, respectively, for the single ASCT subgroup (2984 patients) versus 98.3% and 79.4% respectively for the tandem ASCT subgroup (291 patients).

### Consolidation therapy

Only 205 patients (5.3%) received consolidation. The consolidation regimen mostly often consisted of a PI combined with an IMiD (in 72%). Specific consolidation regimens included: lenalidomide + bortezomib +/- steroids in 55.6%, lenalidomide +/- steroids in 21%, lenalidomide + another PI (ixazomib) +/- steroids in 12.2%, carfilzomib + lenalidomide + steroids in 4.4%, bortezomib + steroids +/- cyclophosphamide in 3.9%, and other regimen in 2.9%. Overall, regardless of the consolidation regimen used, the ORR was 90.2%, with a ≥VGPR rate of 76.6% in the 205 patients. The median duration of consolidation therapy was 63 days (lower (25%) and upper (75%) quartiles: 56 days and 106 days). Most of the patients receiving consolidation also received maintenance therapy (90.7%).

### Maintenance therapy

In the entire cohort, 2061 patients (54%) received maintenance therapy. The following individual regimens were: lenalidomide +/- steroids in 78.3%, bortezomib +/- steroids in 2.9%, lenalidomide + bortezomib +/- steroids in 1.7%, another PI (ixazomib) +/- steroids in 1.6%, lenalidomide + another PI (ixazomib) +/- steroids in 5.5% and thalidomide +/- steroids in 8.5%. In the 1614 evaluable patients receiving maintenance with lenalidomide +/- steroids, the best ORR was 95.5%, with a ≥VGPR rate of 89.7%

Most of the maintenance patients receiving a combination of lenalidomide and bortezomib +/- steroids (33/36 evaluable patients, 91.7%) or bortezomib +/- steroids (37/47 evaluable patients, 78.7%) were high-risk by cytogenetic classification. Conversely, for the lenalidomide +/- steroids subgroup, only 28.5% were considered as high-risk (278/976 evaluable patients) while 71.5% were standard-risk (698/976 evaluable patients) (cytogenetic risk data was unknown for the remaining patients). Of note, 278 patients in the high-risk cytogenetics subgroup (62.8%) received a lenalidomide-based maintenance regimen (lenalidomide +/- steroids).

Of the 2061 patients that received any maintenance regimen, 1173 patients (56.9%) had already discontinued treatment at time of study datalock; the median duration on maintenance therapy for these individuals was 15.7 months (0.1–154 months). For evaluable patients receiving any maintenance therapy (926 patients), median treatment duration was 17.6 months (0.1–122.5 months). For the 859 patients (41.7%) that discontinued maintenance with lenalidomide + /- steroids, the median duration of maintenance was 17.8 months (0.1–122.6 months). The reasons for discontinuation of most common maintenance therapy are summarized in Table 4. Disease progression was the most common reason for discontinuation of maintenance (61.0%), and the median maintenance duration for these 716 patients was 18.8 months (0.5–154 months); the median duration of maintenance was 9.4 months (0.1–122.6 months) for the 363 patients (31.5%) who discontinued for toxicities, secondary malignancies, and death.

**Table 4:** Reasons for discontinuation of most common maintenance therapy.

Reasons for discontinuation	R/RD, *n* (%)	V/VD, *n* (%)	RV/RVD, *n* (%)
	(*n* = 1614)	(*n* = 59)	(*n* = 36)
Progressive disease	544 (33.7)	28 (47.5)	21 (58.3)
No evidence of progressive disease	710 (44.0)	17 (28.8)	12 (33.3)
Toxicity	225 (13.9)	5 (8.5)	2 (5.6)
Patient refusal of treatment	24 (1.5)	0 (0)	1 (2.8)
Secondary malignancy	23 (1.4)	1 (1.7)	0 (0)
Death	19 (1.2)	2 (3.4)	0 (0)
Lost to follow up	41 (2.5)	0 (0)	0 (0)
Treatment completed	2 (0.1)	3 (5.1)	0 (0)
Doctors’ decision	8 (0.5)	2 (3.4)	0 (0)
Unknown	18 (1.2)	1 (1.7)	0 (0)

*R* lenalidomide, *D* dexamethasone, *V* bortezomib.

### Outcomes

Outcomes according to different ASCT components and cytogenetic risk category are summarized in Table 5.

**Table 5:** Treatment and outcomes in 3821 newly diagnosed ASCT patients.

Treatments	mPFS	*p*-value	mOS	*p*-value
**No. of induction regimens**				
1 regimen (*n* = 3445)	36.2 (34.1–38.1)	0.001	126 (120–138)	0.011
2 regimens (*n* = 376)	27.9 (24.3–33.9)		118 (93.7 NRY)	
**No. of ASCTs**				
Single (*n* = 3507)	35.4 (33.6–37.3)	0.62	126 (120–138)	0.23
Tandem (*n* = 314)	34.2 (30.2–47.5)		113 (84.4-NRY)	
**Single ASCT and maintenance given**				
Standard-risk (*n* = 769)	54.7 (47.9–60.3)	0.002	164.2 (158.4, NRY)	<0.001
High-risk (*n* = 290)	36.7 (28.4–47.7)		99.8 (80.1, NRY)	
**Tandem ASCT and maintenance given**				
Standard-risk (*n* = 29)	47.4 (21.9–73.5)	0.4	NRY (NRY-NRY)	0.1
High-risk (*n* = 153)	35.3 (26.2–47.8)		84.1 (69.7-133.0)	
No. of ASCTs—High-risk*				
Single (*n* = 503)	25.1 (23.1–30.5)	0.01	91.5 (75.8-106)	0.008
Tandem (*n* = 201)	35.4 (26.2–52.4)		84.4 (70.3-NRY)	
**No. of ASCTs—Standard-risk**				
Single (*n* = 1178)	46.5 (43.1-51.3)	<0.001	158.6 (147.1-NRY)	/
Tandem (*n* = 37)	33.8 (29.4-NRY)		NRY (NRY–NRY)	
**No. of ASCTs—Unknown-risk**				
Single (*n* = 1825)	32.7 (31.2–35.4)	0.23	120.6 (110.6-134)	0.02
Tandem (*n* = 76)	34.2 (22.9–42.8)		113.3 (73.6-NRY)	
**Consolidation given**				
No (*n* = 3616)	34.4 (32.9–36.6)	<0.001	124 (118-135)	0.07
Yes (*n* = 205)	55.3 (43.9–76.0)		NRY (114-NRY)	
**No Consolidation given**				
Standard-risk (*n* = 1117)	45.9 (41–50.8)		164.4 (147.1-NRY)	
High-risk (*n* = 630)	26.6 (23.6–32)	<0.001	87.9 (76.4-103)	<0.001
Unknown risk (n = 1868)	32.3 (30.8–34.6)		119.5 (110.4-128)	
**Consolidation given**				
Standard-risk (*n* = 98)	52.2 (43.9–78.3)		NRY (114-NRY)	
High-risk (*n* = 74)	42.4 (26.7-NRY)	<0.001	NRY (68.5-NRY)	<0.001
Unknown risk (*n* = 33)	86.9 (54.8-NRY)		NRY (NRY-NRY)	
**Maintenance given (any)**				
Yes (*n* = 2061)	48.8 (46.1–53.5)	<0.001	159 (142-NRY)	<0.001
No (*n* = 1760)	24.5 (23.2–26.7)		105 (97.2-115)	
**No Maintenance given**				
Standard-risk (*n* = 417)	28.9 (23.7–33.7)		129.8 (107.9-NRY)	
High-risk (*n* = 261)	13.4 (11.9–18.8)	<0.001	63.5 (52.3-82.9)	<0.001
Unknown risk (*n* = 1082)	25.7 (24–28.1)		105.1 (97.2-116.5)	
**Maintenance given (any)**				
Standard-risk (*n* = 798)	54.7 (47.9–60.3)		164.4 (158.6-NRY)	
High-risk (*n* = 443)	36.6 (32.2–47.5)	<0.001	97.4 (87.9-NRY)	<0.001
Unknown risk (*n* = 820)	48.7 (44.2–58.7)		158.5 (136.6-NRY)	
**No. of induction regimens and no maintenance**				
1 regimen (*n* = 1602)	32.0 (30.0–34.1)	0.021	108 (100.1-117)	<0.001
2 regimens (*n* = 158)	23.5 (20.1–33.4)		75.8 (39.6-118)	
**1 induction and no maintenance**				
Standard-risk (*n* = 377)	30.4 (25.3–35.4)	0.005	129.0 (107.5-164.2)	0.1
High-risk (*n* = 224)	16.1 (12.2–20.4)		68.0 (51.8-91.9)	
**2 inductions and no maintenance**				
Standard-risk (*n* = 40)	13.5 (4.0–24.4)	0.3	75.6 (27.0-NRY)	0.5
High-risk (*n* = 37)	11.4 (5.4–16.4)		41.3 (23.1-91.0)	
**No. of induction regimens & maintenance**				
1 regimen (*n* = 1843)	55.3 (52.1–60.5)		158.6 (142.5-NRY)	
2 regimens (*n* = 218)	51.1 (44.7 70.7)	0.11	NRY (135.1 NRY)	0.13
**1 induction and maintenance**				
Standard-risk (*n* = 726)	55.1 (48.4–61.5)	0.002	164.0 (158.4-NRY)	<0.001
High-risk (*n* = 391)	35.6 (30.9–47.3)		97.0 (81.2-NRY)	
**2 inductions and maintenance**				
Standard-risk (*n* = 72)	47.1 (27.3–57.1)	0.1	NRY (85.3-NRY)	0.001
High-risk (*n* = 52)	36.5 (22.6–60.9)		84.1 (59.9-NRY)	

*Risk characterization based on fluorescent in situ hybridization (FISH). High-risk: presence of any one or combination of del17p, t(4:14), t(14:16); Standard-risk: negative for del17p, t(4:14) and t(14:16).

The median PFS and OS for all patients with a single ASCT as frontline therapy were 35.4 months (95% CI 33.7-37.3) and 126 months (95% CI 120–138), respectively. In high-risk FISH patients, the median PFS for single vs tandem ASCT was 25.1 vs 35.4 months (*p* = 0.01) and median OS, 91.5 vs 84.4 months (*p* = 0.008), respectively. For those without high-risk FISH, median PFS and OS for single vs tandem ASCT were, respectively, 46.5 vs 33.8 months (*p* < 0.001) and 158.6 vs NYR (not yet reached). For patients with standard-risk MM receiving a single ASCT and any type of maintenance therapy, the median PFS and median OS were 54.7 months (95% CI 47.9–60.3) and 164.2 months (95% CI 158.4-NYR). For patients with high-risk MM receiving a tandem ASCT and any type of maintenance therapy, the median PFS and OS were 35.3 months (95% CI 26.2–47.8) and 84.1 months (95% CI 69.7–133). Looking only at the high-risk patients receiving any type of maintenance therapy, the median PFS for single vs tandem ASCT was 36.7 vs 35.3 months (*p* = 0.80) and median OS, 99.8 vs 84.1 months (*p* = 0.70), respectively.

Those given a second induction regimen had significantly inferior outcomes (median PFS 27.9 vs 36.2 months [*p* = 0.001]; median OS 118 vs 126 months [*p* = 0.011]), although when maintenance was used, results were comparable regardless of the number of induction regimens administered (median PFS 55.3 vs 51.1 months [*p* = 0.11]; median OS 158.6 months vs NYR [*p* = 0.13]). For patients receiving any type of maintenance therapy, the median PFS and OS for one vs two induction regimens were, in standard-risk patients, 55.1 vs 47.1 months (*p* = 0.08) and 164 months vs NYR (*p* = 0.90), respectively, and in high-risk patients, 35.6 vs 36.5 months (*p* = 0.80) and 97.0 vs 84.1 months (*p* = 0.50), respectively.

Consolidation patients had a longer median PFS (55.3 vs 34.4 months [*p* = 0.001]), but no significant gain in median OS (*p* = 0.065). For the standard-risk patients, the median PFS was 45.9 months (95% CI 41–50.8) when no consolidation was given versus 52.2 months (95% CI 43.9–78.3) when consolidation was administered (*p* = 0.01). Median OS in this subgroup was 164.4 months (95% CI 147.1-NYR) vs NRY (95% CI 114-NRY), respectively, (*p* = 0.5). For the high risk population, the median PFS was 26.6 months (95% CI 23.6-32) when no consolidation was given versus 42.4 months (95% CI 26.7-NRY) when consolidation was given (*p* = 0.002). Median OS in this subgroup was 87.9 months (95% CI 76.4-103) vs NRY (95% CI 68.5-NRY) respectively (*p* = 0.6).

**Fig. 1:**
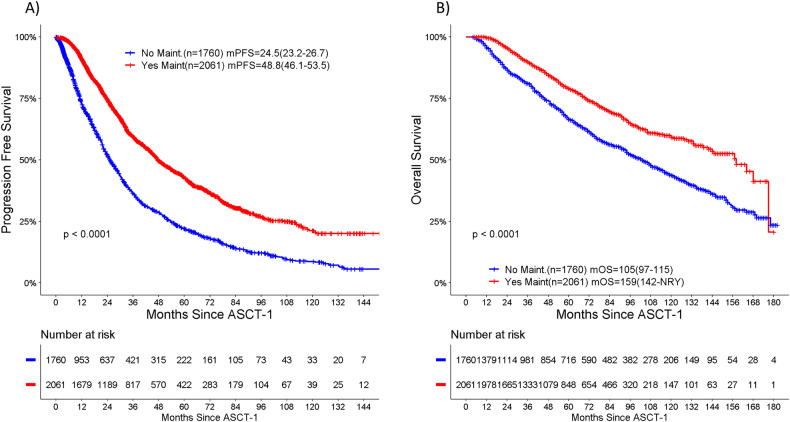
Outcomes in MM patients stratified by any maintenance or no maintenance post-frontline ASCT. **A** PFS. **B** OS.

For the maintenance vs non-maintenance cohorts, respectively, when all types of maintenance were included, the median PFS and median OS were 48.8 vs 24.5 months (*p* < 0.001) and 159 vs 105 months (*p* < 0.001) (Fig. 1). For patients receiving lenalidomide, bortezomib or both (+/- steroids), the median PFS was 53.7 months vs 43.5 months vs 48.2 months, respectively, while the median OS was 159 months vs 115 months vs NYR, respectively. Amongst patients receiving such maintenance, outcomes for patients with known high-risk FISH were compared with those without documented high-risk cytogenetics. The median PFS was 36.6 months (95% CI 32.2-47.5) vs 54.7 months (95% CI 47.9-60.3), and the median OS was 97.4 months (95% CI 87.9-NRY) vs 164.4 months (95% CI 158.6-NRY), respectively. The subgroup of patients with known high-risk FISH and no maintenance therapy did poorly with a median PFS of only 13.4 months (95% CI 11.9–18.8) and a median OS of 63.5 months (95% CI 52.3–82.9). In all risk subgroups, the use of maintenance therapy was associated with better outcomes (*p* < 0.0001). For patients receiving lenalidomide-based maintenance, those who discontinued due to progressive disease had a median PFS of 25 months (95% CI 23.2–27.4) and a median OS of 94 months (95% CI 83–106).

Although some patients received maintenance therapy, including lenalidomide or PIs via mechanisms such as private drug insurance or limited access programs, PFS and OS were also calculated before and after 2013, the year that lenalidomide maintenance until progression was funded throughout Canada and therefore widely accessible (Supplementary Fig. 1). A statistically significant improvement for PFS and OS was identified after 2013 compared to the previous era: median PFS 48.8 versus 24.5 months and OS 159 versus 105 months, respectively (*p* < 0.0001 for both). Thus, universal access to lenalidomide maintenance positively influenced national outcomes of myeloma among transplanted patients.

### Univariable and multivariable analysis for PFS and OS

Table 6 summarizes the results of the univariate and multivariable analysis. In the multivariable analysis for PFS, the disease features significantly associated (*p* < 0.001) with a less favourable PFS included a high beta 2-microglobulin level (HR 1.23 [95% CI 1.10–1.37]) and high-risk cytogenetics (HR 1.56 [95% CI 1.36–1.78]); the administration of ≥2 induction regimens was also associated with an inferior PFS (HR 1.35 [95% CI 1.17–1.56]) while the use of maintenance was correlated with a significantly longer PFS (HR 0.56 [95% CI 0.47–0.57] *p* < 0.001). Multivariable analysis of OS identified increasing age (HR 1.02 [95% CI 1.00–1.28]), LDH > 250 U/L (HR 1.51 [95% CI 1.27–1.78]) and high-risk cytogenetics (HR 1.91 [95% CI 1.60–2.28]) as adverse factors, while patients who received any type of maintenance therapy vs no maintenance experienced a significantly better OS (HR 0.58 [95% CI 0.51–0.67]).

**Table 6:** Univariate and multivariate analysis of factors associated with progression and death according if any maintenance was given or not (*n* = 3821).

Factors		Progression		Death	
		Univariable analysis (HR and 95% CI)^1^	Multivariable analysis (HR and 95% CI)	Univariable analysis (HR and 95% CI)	Multivariable analysis (HR and 95% CI)
**Sex**	Male vs. Female	1.00 (0.92, 1.09)		1.13 (1.00, 1.27)	1.13 (1.00, 1.28)*
**Age at treatment initiation**	Each 1 year increase	1.00 (0.99, 1.01)		1.02 (1.01, 1.03)***	1.02 (1.01, 1.03)***
**Myeloma isotype**	IgG	ref			
	IgA	1.12 (1.00, 1.25)*	1.07 (0.92, 1.24)	1.19 (1.02, 1.38)*	1.18 (1.01, 1.37)*
	Light chain only	0.86 (0.75, 0.99)*	0.83 (0.72, 0.95)**	0.89 (0.73, 1.08)	0.94 (0.77, 1.14)
	Others	1.13 (0.81, 1.58)	1.01 (0.72, 1.42)	1.07 (0.65, 1.75)	0.92 (0.56, 1.53)
	Unknown or missing	1.27 (1.09, 1.49)**	0.98 (0.83, 1.17)	1.40 (1.14, 1.71)**	1.25 (1.00, 1.55)*
**ISS stage**	I	ref			
	II	1.19 (1.06, 1.33)**		1.18 (1.01, 1.39)*	1.05 (0.88, 1.26)
	III	1.49 (1.33, 1.66)***		1.77 (1.52, 2.07)***	1.34 (1.11, 1.63)**
	Unknown or missing	1.30 (1.13, 1.50)**		1.24 (1.01, 1.53)*	1.17 (0.93, 1.45)
**Laboratory results**	Hemoglobin, <=80 vs. >80 g/L	1.39 (1.22, 1.59)***		1.40 (1.17, 1.68)***	
	Hemoglobin Unknown vs. >80 g/L	1.12 (0.94, 1.33)		0.80 (0.61, 1.05)	
	Platelet count <=50 vs. >50 (10^9^/L)	2.02 (1.37, 2.98)***	1.42 (0.96, 2.10)	2.50 (1.57, 3.99)***	1.92 (1.19, 3.08)**
	Platelet count Unknown vs. >50 (10^9^/L)	1.17 (1.05, 1.31)*	0.91 (0.77, 1.06)	0.99 (0.84, 1.16)	0.81 (0.65, 1.02)
	Beta 2-microglobulin >=466.5 vs. <466.5 nmol/L (5.5 mg/L)	1.41 (1.27, 1.56)***	1.23 (1.10, 1.37)***	1.61 (1.40, 1.85)***	
	Beta 2-microglobulin Unknown vs. <466.5 nmol/L (5.5 mg/L)	1.23 (1.10, 1.37)***	1.11 (0.98, 1.25)	1.13 (0.96, 1.33)	
	Albumin <=35 vs. >35 g/L	1.26 (1.15, 1.38)***	1.12 (1.01, 1.23)*	1.31 (1.16, 1.49)***	1.16 (1.00, 1.36)
	Albumin Unknown vs. >35 g/L	1.36 (1.20, 1.54)***	1.07 (0.90, 1.27)	1.13 (0.95, 1.35)	0.95 (0.74, 1.21)
	Calcium >=2.65 vs. <2.65 mmol/L	1.33 (1.18, 1.50)***	1.23 (1.09, 1.39)**	1.51 (1.29, 1.77)***	1.27 (1.07, 1.51)**
	Calcium Unknown vs. <2.65 mmol/L	1.26 (1.12, 1.40)***	1.05 (0.89, 1.24)	1.17 (1.00, 1.37)	1.32 (1.03, 1.68)*
	Creatinine >177 umol/L vs. <=177	1.23 (1.09, 1.38)***		1.50 (1.29, 1.75)***	1.10 (0.92, 1.32)
	Creatinine Unknown vs. <=177 umol/L	1.16 (0.99, 1.36)		0.78 (0.60, 1.01)	0.70 (0.52, 0.95)*
	LDH, >250 U/L vs. <=250	1.38 (1.21, 1.56)***	1.25 (1.09, 1.42)**	1.66 (1.41, 1.96)***	1.51 (1.27, 1.78)***
	LDH Unknown vs. <=250 U/L	1.31 (1.19, 1.43)***	1.10 (0.99, 1.22)	1.19 (1.04, 1.36)*	1.06 (0.90, 1.25)
**Cytogenetic risk classification**	Standard-risk	ref			
	High-risk^2^	1.56 (1.37, 1.77)***	1.56 (1.36, 1.78)***	2.04 (1.71, 2.43)***	1.91 (1.60, 2.28)***
	Unknown or missing	1.34 (1.22, 1.48)***	1.10 (0.99, 1.22)	1.46 (1.27, 1.69)***	1.28 (1.10, 1.49)**
**Induction regimens**	Two or more	1.29 (1.12, 1.48)***	1.35 (1.17, 1.56)***	1.29 (1.06, 1.56)*	1.27 (1.05, 1.54)*
**ASCT type**	Single	ref			
	Tandem vs. single	1.04 (0.88, 1.24)	0.95 (0.79, 1.15)	1.29 (1.06, 1.56)*	
**Consolidation**	Yes vs. no	0.65 (0.53, 0.79)***	0.79 (0.64, 0.96)*	0.78 (0.59, 1.02)	
**Maintenance**	**Yes vs. no**	0.52 (0.48, 0.57)***	0.52 (0.47, 0.57)***	0.59 (0.52, 0.67)***	0.58 (0.51, 0.67)***

*FISH* Fluorescent in situ hybridization, *HR* Hazard ratio, *ASCT* Autologous stem cell transplant.

1. Note that variables with HR > 1 were associated with higher risk of progression or death and HR < 1 indicated lower risk of progression or death.

2. High-risk was defined by the presence by FISH of any one or combination of del17p, t(4:14), t(14:16).

*p < 0.05, **p < 0.01, ***p < 0.001.

Looking at only maintenance with lenalidomide +/- steroids versus no maintenance as predictors, the multivariate analysis was still significant for the risk of progression (HR 0.48 (95% CI, 0.43–0.53)) as well as the risk of death (HR 0.53 (95% CI, 0.46–0.62)), favoring maintenance therapy in lowering those risks. The same tendency was observed when looking at other maintenance regimens versus no maintenance with a HR of 0.67 (95% CI, 0.58–0.77) for the risk of progression and of 0.73 (95% CI, 0.60–0.89) for the risk of death, also favouring maintenance therapy (Supplementary Table 1).

## Discussion

This large real-world study from the national CMRG database demonstrates that with integration of bortezomib, lenalidomide and maintenance therapy into the first-line treatment sequence in transplant-eligible patients, the median OS currently exceeds 10 years, except in high-risk patients. This illustrates nicely the progress made in the care of Canadian multiple myeloma patients in the last decade.

One strength of this CMRG database’s data is the long-term follow-up of a large number of patients treated with a uniform induction regimen, mostly CyBorD. On the other hand, only 1.6% of the patients received an induction therapy with combination of a PI and an IMiD, since this combination (i.e., RVD) as induction therapy pre-ASCT is not widely accessible in Canada. As discussed below, RVD has emerged as the newer standard of care for induction therapy. The current results primarily serve as Canadian benchmarks for comparison with newer approaches in this patient population as well as provide expectations for patients treated with CyBorD either in jurisdictions where RVD is not accessible or for specific reasons.

Our analysis also highlights, in a real-world setting, the contribution of post-ASCT maintenance, particularly lenalidomide-based maintenance. Indeed, in our overall study, patients receiving lenalidomide maintenance or no maintenance, experienced a median PFS of 53.7 months vs 24.5 months, while the median OS was 159 months vs 105 months, respectively, with a median follow-up from induction to end of last follow-up of 50.1 months. Of note, one of the most common reasons for stopping maintenance was disease progression, with only toxicity accounting for 13.9% discontinuation in the lenalidomide +/- steroid group, and even lower in the other groups. The rates of patient or physician withdrawal of maintenance and cessation due secondary malignancy were extremely low. One potential explanation for compliance with maintenance was emphasis placed on side-effect management, including frequent dose reductions as reported in other Canadian reports of lenalidomide maintenance post-ASCT [8, 10].

Our data compares favourably with the meta-analysis of McCarthy and al [4], which showed a median PFS of 23.5 months for the placebo/observation group vs 52.8 months for the lenalidomide group, and a median OS of 86 months vs NRY, respectively, at a median follow-up time of 79.5 months for all surviving patients. These patients had received a variety of induction regimen in the setting of clinical trials. However, in the recently published DETERMINATION study [11], the median PFS was 67.5 months in the transplantation group, and for the high-risk patients receiving transplantation, 55.5 months. Overall, these patients in the transplantation arm had a median age of 55 years, they all received a lenalidomide and bortezomib-based regimen as induction and consolidation therapy, and lenalidomide maintenance post-ASCT was planned until disease progression, with a median duration of 41.5 months. Even keeping in mind that the outcomes were calculated from the time of randomization to RVD induction, after receiving 1 cycle of RVD, while our analysis was calculated from a timepoint later in the course (i.e., at the time of ASCT), the median PFS reported in the DETERMINATION trial was longer than in our experience, and in the previous meta-analysis. Many factors could explain the shorter PFS in our analysis, most likely the fact that our real-world data included patients with medical complications such as renal compromise and severe cytopenias, with an older median age. We also included those requiring a second line induction or undergoing tandem ASCT. Importantly, the DETERMINATION patients all received the currently preferred PI plus IMiD induction as well as similar consolidation, while our Canadian patients predominantly received only bortezomib-based induction regimens; consolidation was given to only a minority of patients, and the median duration of maintenance therapy was shorter in our cohort. Joseph et al. [12] also retrospectively described the outcomes for 1000 patients with newly diagnosed multiple myeloma receiving an induction regimen of lenalidomide, bortezomib and dexamethasone; while some standard-risk patients continued on RVD alone, 751 proceeded to up-front ASCT. Among all patients, 753 received maintenance, which consisted of lenalidomide in 600. When results were calculated from the date of diagnosis, maintenance patients experienced a median PFS of 65.5 months and median OS of 129.8 months. The median duration of maintenance therapy in this study for patients with up-front ASCT was 57 months. This data again shows the benefit on PFS of an induction regimen combining a PI and an IMID over a bortezomib-based only induction, as well as the impact of duration of maintenance therapy. On the other hand, these single-centre results with outcomes measured from a different starting point are challenging to compare with our database results, and the median OS was less in this cohort compared to ours, revealing the impact of other important factors on OS, like sequencing of therapies.

It was of particular clinical interest to demonstrate that the negative impact of needing a second-line induction therapy on PFS and OS was abrogated by maintenance therapy. For high-risk patients, although our data shows, like Joseph et al’s data [12], maintenance therapy significantly improved outcomes, it does not completely mitigate the negative impact of high-risk cytogenetics, and additional strategies are required to better optimize results in this subgroup since the outcomes remain inferior to the standard-risk patients.

This study confirmed that post-ASCT consolidation therapy was not widely used in Canada, as only about 5%, received consolidation, most likely attributable to lack of public funding in most jurisdictions. This strategy, in standard-risk as well as in high-risk patients, did show a better median PFS, but no significant gain in OS.

The use of tandem ASCT in high-risk patients has been a common practice in several Canadian centres. This study showed a significant PFS benefit for tandem ASCT in high-risk patients, but surprisingly, median OS was better with a single ASCT. A decreased effect on survival from the tandem procedure itself has not been described previously. Potential reasons for this finding include an undetected selection bias for those with the highest risk of an eventual aggressive relapse to undergo tandem procedures. For example, we did not capture other adverse features such as extramedullary disease or circulating plasma cell counts <20% that would now reclassify patients as having plasma cell leukemia; if the high-risk FISH criteria were not present or not measured in such patients, they would be classified as standard-risk in this study. Not all centres performed all 3 high-risk FISH abnormalities during the 15-year period encompassed by this analysis and many centres have only recently looked for other important cytogenetic aberrations, such as 1q21 amplification. Our patients were not further classified as ultra high-risk vs high-risk. Efforts to further refine high-risk using more sophisticated molecular panels and other platforms are desirable to best interpret our observations.

Our study also interestingly shows, in the high-risk subgroup, that the benefit of a tandem over a single ASCT was not statistically significant when maintenance was given. In the EMN02/HO95 study [1], for patients with high-risk cytogenetic, median PFS was 46.0 months with double ASCT vs 26.7 months with single ASCT (*p* = 0.062) and the 5-year OS was, respectively, 61.3% vs 54.7% (*p* = 0.32). A benefit for tandem ASCT was only seen for the subgroup of patients with del17p. The long-term follow-up data from the BMT CTN 0702 (STaMINA) trial showed conflicting results with a 6-year PFS in high-risk patients as treated analysis of 43.6% for double ASCT vs 26% for single ASCT (*p* = 0.03) [13]. However, in the intent-to-treat analysis, no difference in PFS or OS was shown. This data shows that treatment options in high-risk patients remain to be optimally defined and that definitive evidence for the utility of tandem transplantation in high-risk patients is lacking.

Our study has limitations, notably its retrospective nature. Data should therefore be interpreted with caution. Another important limitation is that nowadays, induction therapy before ASCT combines a PI and an IMID, and thus generalization of our results to this modern era population is not possible as our most common induction regimen was CyBorD. However, our findings remain relevant for patients who do not have access to RVD as induction therapy and will receive CyBorD instead (e.g. patients with severe renal insufficiency, allergy or intolerance to lenalidomide, no access to lenalidomide for funding purposes).

The issue of missing data should be considered. Our real-world analysis found that information on del17p, t(4;14) and t(14;16) was missing or unknown in 50% of the entire cohort. FISH data was not available or done uniformly across Canada particularly in the earlier years of this study. Indeed, in 2007, cytogenetic risk information was unknown in 77.4% of the patients versus 33.3% in 2021, showing improvement over the years. Similarly, LDH values were missing in 20–40% of individuals in the initial years of the database, decreasing to 12–15% more recently. In turn, the R-ISS score was also unavailable in a significant percentage. Of note, the large Joseph retrospective study [12] also lacked R-ISS in over half of patients, highlighting the challenge of capturing all the desired information outside of a formal prospective study. Nevertheless, the lack of complete risk data, including key FISH features, highlights the need for cautious interpretation of results stratified by cytogenetics.

Finally, real-world database analyses cannot capture all of the different variables included in a clinician’s decision to choose for one treatment option versus another. Such features may involve the use of one or two transplants, specific agent selected for maintenance, maintenance duration and patient decisions, inevitably leading to bias in the interpretation of the data.

Nevertheless, this large study, covering 15 years in the real-world setting, demonstrates that the integration of bortezomib and lenalidomide into the transplant sequence produces a median OS of >10 years in most ASCT patients. Even though recent studies using RVD induction demonstrate a longer PFS and OS and RVD is considered the preferred induction regimen, CyBorD induction is still used in many countries and in the setting of renal compromise or poor blood counts. Our study also highlights the contribution of post-ASCT maintenance, particularly lenalidomide given until progression, in multiple subgroups, including those with and without high-risk MM as well as those receiving a second induction. In the modern era of maintenance therapy, the role of tandem ASCT in high-risk patients still needs to be better defined. Finally, these results serve as benchmarks for comparison with future outcomes resulting from the recent public funding for RVD induction in Canada as well as the newer immunotherapies being introduced into first-line therapy in transplant-eligible patients.

### Supplementary information

Supplemental Material

## Data Availability

The datasets generated during and/or analysed during the current study are not publicly available due to privacy laws but access is available through the corresponding author on reasonable request.
