# Supplementary material for: Real-world results of autologous stem cell transplantation in newly diagnosed multiple myeloma: a report from the Canadian Myeloma Research Group database
Source: Blood Cancer J. 2023 Sep 5;13(1):137. doi: 10.1038/s41408-023-00905-8 (PMC10480201; doi:10.1038/s41408-023-00905-8)

**SUPPLEMENTAL DATA**

**Supplemental Table 1.** Univariate and multivariate analysis of factors associated with progression and death according if lenalidomide-based or other type of maintenance regimen was given or not (n = 3821)

| **Factors** | | **Progression** | | **Death** | |
| --- | --- | --- | --- | --- | --- |
|  |  | **Univariable analysis (HR and 95% CI) ^1^** | **Multivariable analysis (HR and 95% CI)** | **Univariable analysis (HR and 95% CI)** | **Multivariable analysis (HR and 95% CI)** |
| **Sex** | Male vs. Female | 1.00(0.92, 1.09) |  | 1.13(1.00, 1.27) | 1.14 (1.01, 1.29) * |
| **Age at treatment initiation** | Each one year increase | 1.00 (0.99, 1.01) |  | 1.02 (1.01, 1.03) *** | 1.02 (1.01, 1.03) *** |
| **Myeloma isotype** | IgG | ref | | | |
|  | IgA | 1.12 (1.00, 1.25) * | 1.09 (0.98, 1.22) | 1.19 (1.02, 1.38) * | 1.18 (1.01, 1.37) * |
|  | Light chain only | 0.86 (0.75, 0.99) * | 0.83 (0.72, 0.95) ** | 0.89 (0.73, 1.08) | 0.94 (0.77, 1.15) |
|  | Others | 1.13 (0.81, 1.58) | 1.02 (0.72, 1.43) | 1.07 (0.65, 1.75) | 0.94 (0.57, 1.56) |
|  | Unknown or missing | 1.27 (1.09, 1.49) ** | 0.98 (0.83, 1.16) | 1.40 (1.14, 1.71) ** | 1.25 (1.01, 1.55) * |
| **ISS stage** | I | ref | | | |
|  | II | 1.19 (1.06, 1.33) ** |  | 1.18 (1.01, 1.39) * | 1.05 (0.88, 1.26) |
|  | III | 1.49 (1.33, 1.66) *** |  | 1.77 (1.52, 2.07) *** | 1.36 (1.12, 1.64) ** |
|  | Unknown or missing | 1.30 (1.13, 1.50) ** |  | 1.24 (1.01, 1.53) * | 1.18 (0.95, 1.48) |
| **Laboratory results** | Hemoglobin, <=80 vs. >80 g/L | 1.39 (1.22, 1.59) *** |  | 1.40 (1.17, 1.68) *** |  |
|  | Hemoglobin Unknown vs. >80 g/L | 1.12 (0.94, 1.33) |  | 0.80 (0.61, 1.05) |  |
|  | Platelet count <= 50 vs. >50 (10^9^/L) | 2.02 (1.37, 2.98) *** | 1.44 (0.97, 2.14) | 2.50 (1.57, 3.99) *** | 1.93 (1.20, 3.10) ** |
|  | Platelet count Unknown vs. >50 (10^9^/L) | 1.17 (1.05, 1.31) * | 0.90 (0.76, 1.05) | 0.99 (0.84, 1.16) | 0.80 (0.64, 1.01) |
|  | Beta 2-microglobulin >=466.5 vs. <466.5 nmol/L | 1.41 (1.27, 1.56) *** | 1.23 (1.10, 1.38) *** | 1.61 (1.40, 1.85) *** |  |
|  | Beta 2-microglobulin Unknown vs. <466.5 nmol/L | 1.23 (1.10, 1.37) *** | 1.12 (0.99, 1.26) | 1.13 (0.96, 1.33) |  |
|  | Albumin <=35 vs. >35 g/L | 1.26 (1.15, 1.38) *** | 1.12 (1.02, 1.24) * | 1.31 (1.16, 1.49) *** | 1.17 (1.01, 1.37) * |
|  | Albumin Unknown vs. >35 g/L | 1.36 (1.20, 1.54) *** | 1.07 (0.90, 1.27) | 1.13 (0.95, 1.35) | 0.95 (0.74, 1.21) |
|  | Calcium >=2.65 vs. <2.65 mmol/L | 1.33 (1.18, 1.50) *** | 1.22 (1.08, 1.38) ** | 1.51 (1.29, 1.77) *** | 1.26 (1.07, 1.50) ** |
|  | Calcium Unknown vs. <2.65 mmol/L | 1.26 (1.12, 1.40) *** | 1.06 (0.89, 1.25) | 1.17 (1.00, 1.37) | 1.32 (1.04, 1.69) * |
|  | Creatinine > 177 umol/L vs. <=177 | 1.23 (1.09, 1.38) *** |  | 1.50 (1.29, 1.75) *** | 1.08 (0.90, 1.29) |
|  | Creatinine Unknown vs. <=177 umol/L | 1.16 (0.99, 1.36) |  | 0.78 (0.60, 1.01) | 0.70 (0.52, 0.95) * |
|  | LDH, > 250 U/L vs. <=250 | 1.38 (1.21, 1.56) *** | 1.27 (1.11, 1.44) ** | 1.66 (1.41, 1.96) *** | 1.53 (1.29, 1.81) *** |
|  | LDH Unknown vs. <= 250 U/L | 1.31 (1.19, 1.43) *** | 1.12 (0.99, 1.25) | 1.19 (1.04, 1.36) * | 1.08 (0.91, 1.27) |
| **Cytogenetic risk classification** | Standard-risk | ref | | | |
|  | High-risk ^2^ | 1.56 (1.37, 1.77) *** | 1.51 (1.32, 1.73) *** | 2.04 (1.71, 2.43) *** | 1.85 (1.54, 2.21) *** |
|  | Unknown or missing | 1.34 (1.22, 1.48) *** | 1.08 (0.97, 1.19) | 1.46 (1.27, 1.69) *** | 1.25 (1.08, 1.46) ** |
| **Induction regimens** | Two or more | 1.29 (1.12, 1.48) *** | 1.35 (1.17, 1.56) *** | 1.29 (1.06, 1.56) * | 1.27 (1.05, 1.54) * |
| **ASCT type** | Single | ref | | | |
|  | Tandem vs. single | 1.04 (0.88, 1.24) | 0.90 (0.75, 1.09) | 1.29 (1.06, 1.56) * |  |
| **Consolidation** | Yes vs. no | 0.65 (0.53, 0.79) *** | 0.77 (0.62, 0.94) * | 0.78 (0.59, 1.02) |  |
| **Maintenance**  **regimens** | **R/RD vs. no maintenance** | 0.48 (0.43, 0.52) *** | 0.48 (0.43, 0.53) *** | 0.53 (0.47, 0.61) *** | 0.53 (0.46, 0.62) *** |
|  | **Other regimens vs. no maintenance** | 0.69 (0.60, 0.78) *** | 0.67 (0.58, 0.77) *** | 0.76 (0.63, 0.92) ** | 0.73 (0.60, 0.89) ** |

Abbreviations: FISH, Fluorescent in situ hybridization; HR, Hazard ratio; ASCT, Autologous stem cell transplant; R, lenalidomide; RD, lenalidomide+dexamethasone.

1. Note that variables with HR > 1 were associated with higher risk of progression or death and HR < 1 indicated lower risk of progression or death.

2. High-risk was defined by the presence by FISH of any one or combination of del17p, t(4:14), t(14:16).
*p<0.05 **p<0.01 ***p<0.001

**Supplemental Figure 1.** Outcomes in MM patients stratified by before and after 2013. A) PFS B) OS

A)


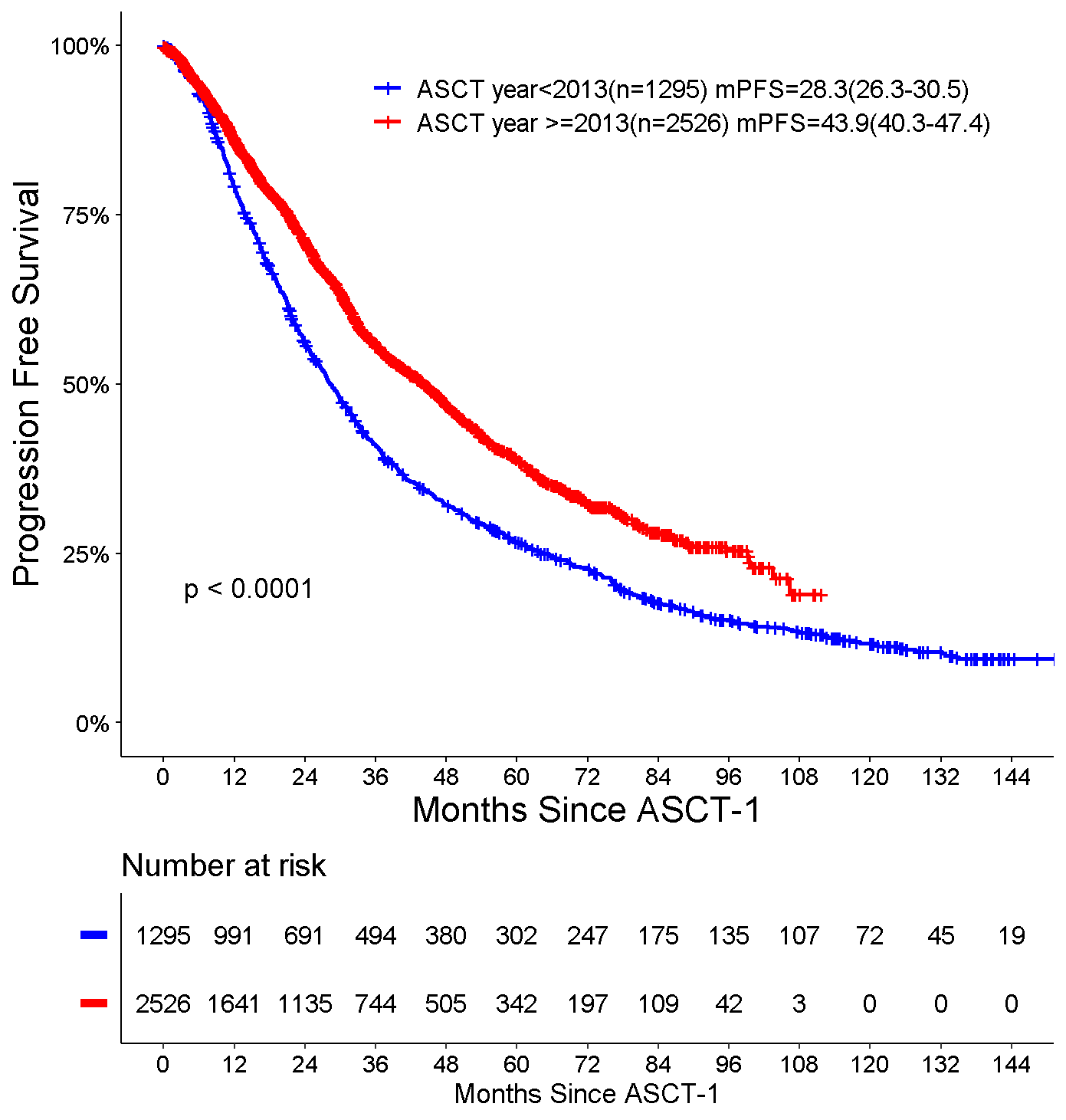


B)


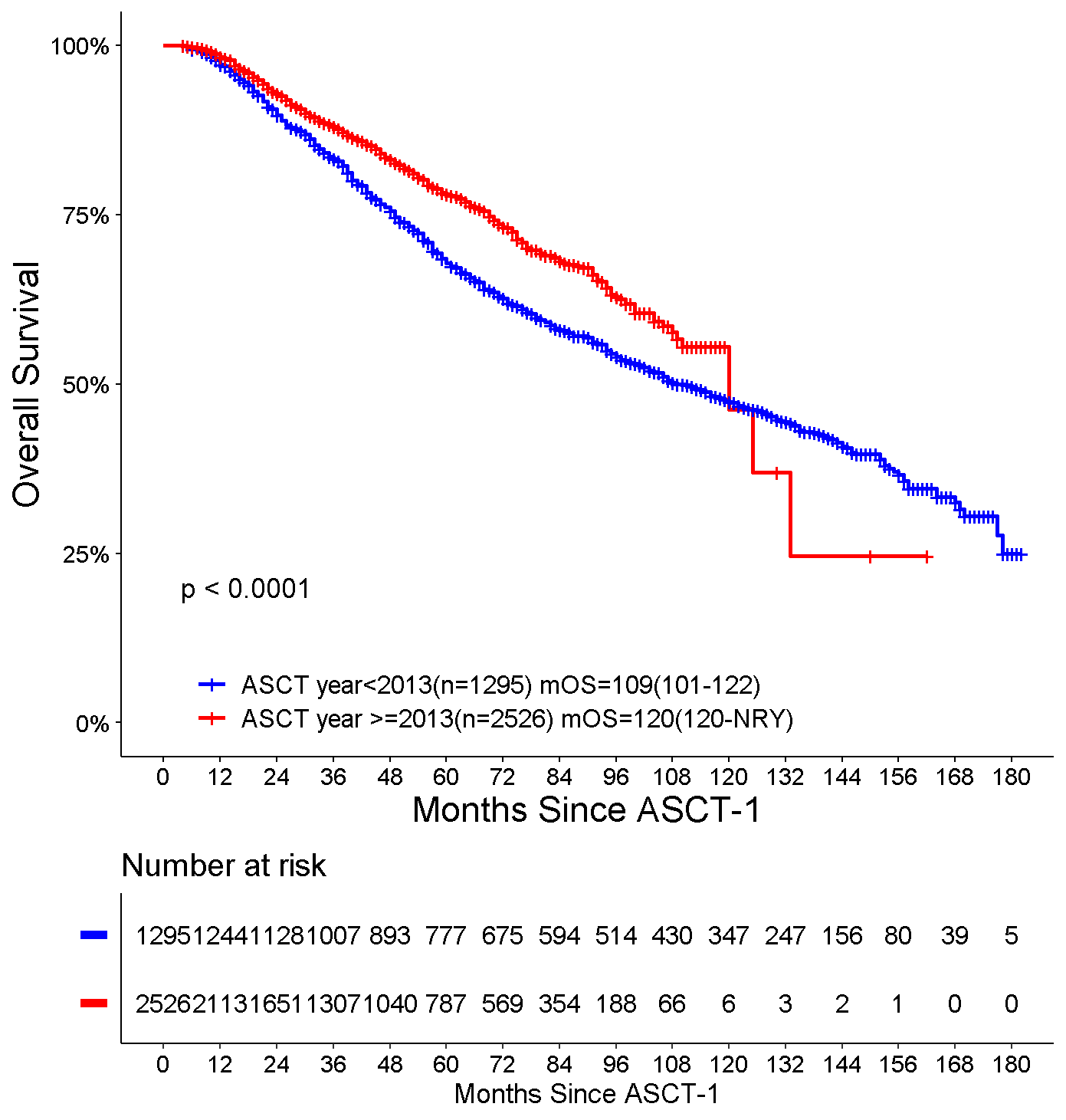

Supplement: Supplementary file 1 — Supplemental Material [file 41408_2023_905_MOESM1_ESM.docx]
